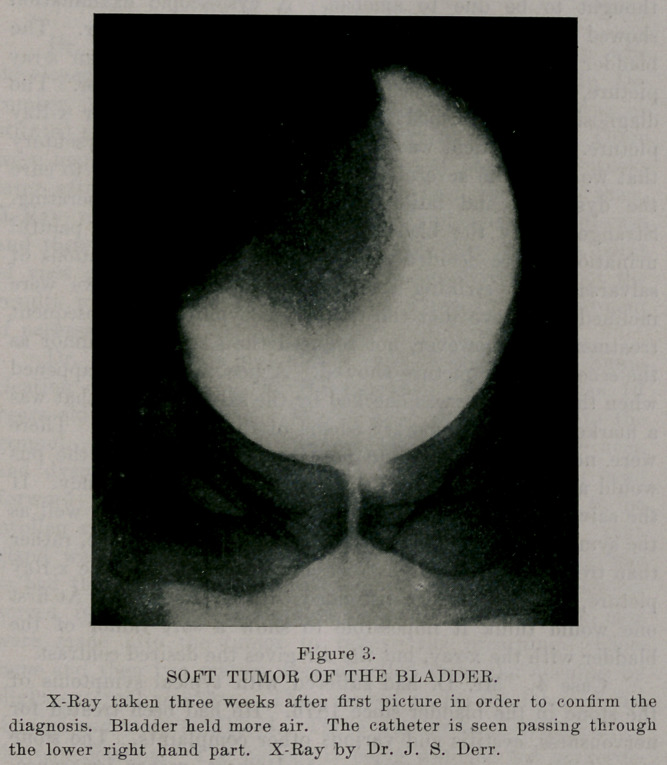# The Diagnosis of Surgical Affections of the Kidney and Bladder

**Published:** 1914-09

**Authors:** Edgar G. Ballenger, Omar F. Elder

**Affiliations:** Atlanta; Atlanta


### THE DIAGNOSIS OF SURGICAL AFFECTIONS OF THE KIDNEY AND BLADDER.

By Edgar G. Ballenger, M. D., and Omar F. Elder, M. D.,
Atlanta.

While remarkable advances have been made in the diagnosis of renal and bladder affections, unfortunately the application of these methods is largely limited to those who confine their work to this branch of surgery. The good to patients is therefore minimized and they still must submit frequently to archaic remedies, with continued disability and perhaps suffering until of their own volition or the insistance of their friends they consult a diagnostician or a specialist who is particularly interested in this field of medicine. After much waste of time, health and money, the patient finally has a correct diagnosis made, but he often resents the fact that his family physician neglected so long a more thorough examination. The patients do not expect the family physician to take the x-ray picture, to do a cystoscopic examination, to catheterize the ureters, to test with pyelography, to do a functional renal test, or perhaps even to make a microscopic examination for pus, or blood. They do expect and not without reason, that the physician should exercise common sense directing the employment of these measures and not continue the usual remedies indefinitely with negative results. The physician who recognizes his limitation is held in high esteem because the patients feel that he will suggest thorough examinations when they are needed. To wait until the patient goes to another physician who arranges for suitable tests to make a correct diagnosis results in the loss of the patient's confidence.

Merely to demonstrate pus or blood in the urine, even though it be examined most thoroughly by an expert, is not making a diagnosis; unless its source or sources are found as well as the localized cause or causes, the condition can not be said to be diagnosed. The battle is then more than half over. The vast majority of bad results come not from poor operative technic, but from incomplete examinations in making the diagnosis. If in America our diagnoses were equal to our surgical technic, we would far excel all competitors. This applies with particular force to the renal and bladder surgery. To remove a stone in the bladder and leave the urethra obstructed by stricture or prostatic hypertrophy means failure; to operate on one kidney and leave serious disease in the other will likewise give a poor result; to remove the only kidney is a tragedy. Nevertheless these unfortunate occurrances are too frequently seen.

Figure 1.

STONES IN THE KIDNEY.

At operation a shell of a kidney was removed, containing 400 small stones. X-Ray by Dr. J. S. Derr.

Pain, pus and blood are the red flags warning the patients of trouble; their significance and proper interpretation often require cystoscopy, ureteral catheterization, pyelography and x-ray examinations. All of these, interpreted with the clinical history and symptoms, may leave uncertainty at times as to the exact diagnosis. Our understanding however, of the existing condition is much better than if they were not employed. As a rule a certain diagnosis can be made promptly, not only determining the character and location of the disease, but also the separate functional capacity of each kidney. The cystoscopic examination readily reveals pathologic conditions of the bladder; the condition of the ureteral orifices also often directs attention to a tubercular or otherwise diseased kidney. Observation of the flow of blue urine from the ureters after the intravenous injections of indigocarmin, the promptness of the blue color, its intensity, and the ratio of the quantity passed the first half hour and that of the second imparts to us valuable information as to the renal functional activity.

Catheterization of the ureters often enables us to locate the source of the renal pus and blood. Pain is frequently misleading. The phenolsulphonephthalein test with the ureters catheterized shows the functional capacity of each kidney with an unusual degree of accuracy.

The injections of collargol, silver iodide, or argyrol into the ureters followed by an x-ray picture demonstrates abnormalities of the renal pelvis and ureter. This test is not entirely free from disadvantages and should be used with discrimination.

X-ray plates of the bladder and kidney are many times absolutely indispensable.

The following case histories are of interest and show how impossible it would have been to have made a correct diagnosis without the employment of one or more of the above mentioned tests. Many other histories could be reported, but it would unduly prolong the paper.

Case 1. Mrs. A. was referred to us for cystoscopic examination to determine the source of the pus in the urine. At times the quantity of pus would be so enormous that it would on sedimentation equal in volume one-half to one-third of the urine when the quantity of the urine was abo\e normal. At other times the urine would be almost clear, and showed no

Figure 2.

TUMOR OF THE BLADDER.

The bladder was filled with air and then the x-Ray picture was atken. Cystoscopically the tumor appeared to be a papilloma. X-Ray by Derr.

casts, and not more albumin than the pus would account for. She had several attacks of acute cystitis two or three years ago, but remained well until the past few months when the present trouble made its onset. She had been given an intramuscular injection of quinine in the right hip for malaria. This was followed by an abscess at the site of the injection, which burrowed deeply but did not come to the surface. It caused excruciating pain along the sciatic nerve for months and the patient lost flesh until she weighed only 87 pounds. There was an extensive, hard, spindle-shaped swelling of the upper thigh and hip, which, except for the excessive tenderness, appeared to be an osteasarcoma. An x-ray examination, however, was negative except that it showed an apparent softening of the head of the femur. Upon attempting to do a cystoscopic examination great difficulty was experienced in washing the bladder free from pus. There seemed to be an almost inexhaustible supply. Finally we looked into the bladder and saw the pus coming from a small opening on the right side about the size of the mouth of the ureter. Indigocarmin was injected and came through the ureters in a normal manner, the drainage, however, from the third orifice was not stained blue. These facts clearly demonstrated that the pus did not come from a pus-kidney as we had expected, but that it came from an abscess of the hip. This was subsequently confirmed by drainage on the abscess and clearing up of the urine. Dr. Rogers, the surgeon who incised it in Jacksonville, made the following report:

"The abscess lay beneath the gluteus medius and maximus muscles, both of the muscles being extensively infiltrated with inflammatory products, pale and oedematous, evidently forming an unusually firm barrier toward the external exit of pus. After passing through the gluteus medius there was no difficulty in getting into the abscess, which was quite large and there was considerable destruction of the tissue deep in about the sacro-sciatic notch. I was able to demonstrate clearly that the pus was pouring through the sacro-sciatic foramen, and evidently it followed the sciatic nerve into the pelvis and then followed reptroperitoneally below the brim of the pelvis and along the ureter into the bladder."

Case 2. Miss B. suffered for 7 years with recurrent renal colic most severe in character. The urine at times was clear and at other very muddy. At the time of our examination the patient was a confirmed neurasthenic. Much of her complaining had been attributed to neurasthenia. She has passed through the hands of several very well known diagnosticians without a

Figure 3.

SOFT TUMOR OF THE BLADDER.

X-Ray taken three weeks after first picture in order to confirm the diagnosis. Bladder held more air. The catheter is seen passing through the lower right hand part. X-Ray by Dr. J. S. Derr.

correct diagnosis being made. The x-ray picture here shown demonstrated the presence of the stones in the kidney. Catheterization showed the left kidney to be functionally above normal. At operation we removed a shell of a kidney which contained 400 small stones. The patient made an uneventful recovery.

Case 3. Mr. C. had suffered with emebic dysentery for several years. Hematuria later developed and at first was thought to be due to amebae. A cystoscopic examination showed the presence of a large papillomatous tumor. The bladder was filled with air and Dr. J. S. Derr took an x-ray picture, which showed beautifully the size of the tumor. The diagnosis was confirmed three weeks later by another x-Ray picture. The patient was so weakened by the amebic dysentery that we gave him several small injections of salvarsan to cure the dysentery and build up his strength before operating. Strange to say the bladder symptoms, frequent and painful urinations, were decidedly improved by the first injections of salvarsan. So striking was the improvement that we were inclined to believe that the tumor was syphilitic. Subsequent treatment has, however, not reduced the size of the tumor as the second x-Ray picture showed. A peculiar thing happened when the dysentery was checked by the salvarsan, and that was a marked though temporary edema of the lower limbs. There were no casts in the urine nor more albumin than the pus would account for, nor was there any cardiac insufficiency. If the salvarsan does not reduce the size of the tumor as well as the symptoms we will extirpate it, on account of its size, rather than treat it with the high frequency current. This the x-Ray picture, with the bladder distended with air, suggests. At first one would think it impossible to show a soft tumor of the bladder with the x-ray, but the air gives the desired contrast.

Case 4. Mr. D. had suffered with typical symptoms of the stone in the bladder since 1876. He had been treated for nervousness, cystitis and various other complaints. The stone we exhibit is a monument to carelessness, rather ignorance. The latter is more reprehensible than the former. The suffering experienced by this patient during the 38 years with the stone in his bladder has probably been sufficient, if equally subdivided and distributed among his physicians to make considerable better doctors of them. Incidentally the patient states that from a meatotomy in 1878 to glasses fitted in 1913 he has spent $16,000 for incorrect diagnoses.

805 Healey Bldg.

## Figures and Tables

**Figure 1. f1:**
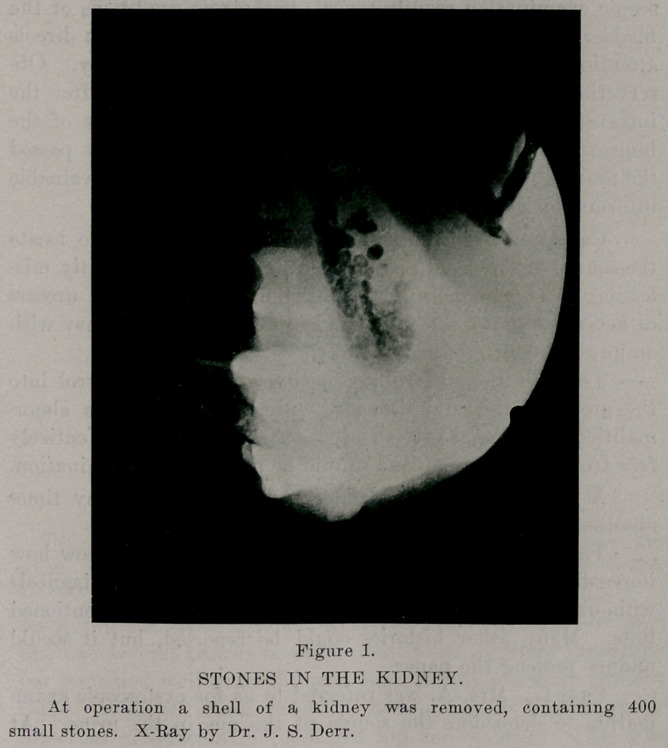


**Figure 2. f2:**
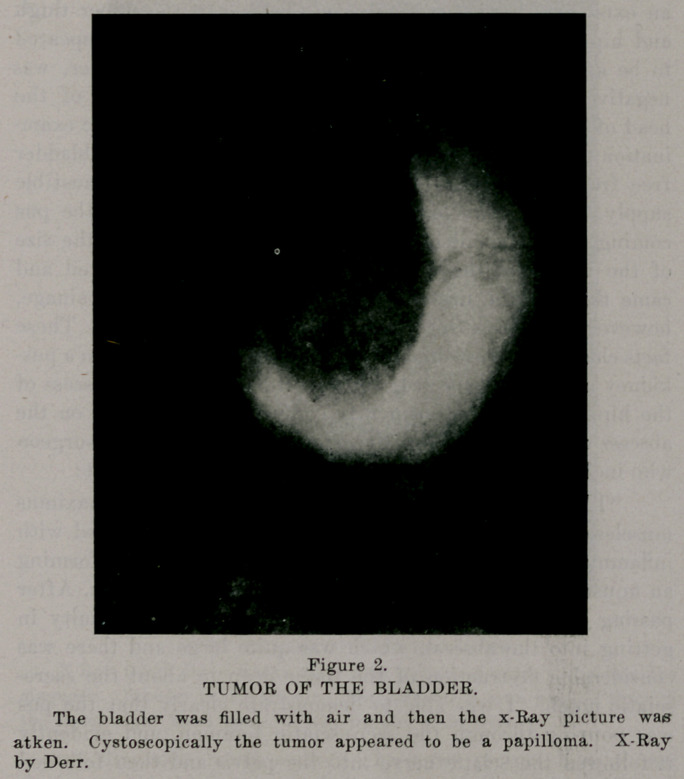


**Figure 3. f3:**